# Thermodynamic Signatures of Structural Transitions and Dissociation of Charged Colloidal Clusters: A Parallel Tempering Monte Carlo Study

**DOI:** 10.3390/molecules27082581

**Published:** 2022-04-16

**Authors:** Frederico V. Prudente, Jorge M. C. Marques

**Affiliations:** 1 Instituto de Física, Universidade Federal da Bahia, Salvador 40170-115, BA, Brazil; 2 CQC–IMS, Department of Chemistry, University of Coimbra, 3004-535 Coimbra, Portugal

**Keywords:** colloids, short-range attraction, long-range repulsion, Bernal spiral, thermodynamic properties

## Abstract

Computational simulation of colloidal systems make use of empirical interaction potentials that are founded in well-established theory. In this work, we have performed parallel tempering Monte Carlo (PTMC) simulations to calculate heat capacity and to assess structural transitions, which may occur in charged colloidal clusters whose effective interactions are described by a sum of pair potentials with attractive short-range and repulsive long-range components. Previous studies on these systems have shown that the global minimum structure varies from spherical-type shapes for small-size clusters to Bernal spiral and “beaded-necklace” shapes at intermediate and larger sizes, respectively. In order to study both structural transitions and dissociation, we have organized the structures appearing in the PTMC calculations by three sets according to their energy: (i) low-energy structures, including the global minimum; (ii) intermediate-energy “beaded-necklace” motifs; (iii) high-energy linear and branched structures that characterize the dissociative clusters. We observe that, depending on the cluster, either peaks or shoulders on the heat–capacity curve constitute thermodynamics signatures of dissociation and structural transitions. The dissociation occurs at T=0.20 for all studied clusters and it is characterized by the appearance of a significant number of linear structures, while the structural transitions corresponding to unrolling the Bernal spiral are quite dependent on the size of the colloidal system.

## 1. Introduction

Interactions involving both short-range attraction and long-range repulsion (SALR) can be observed in a great diversity of systems and phenomena. Proteins in solution [1,2,3,4,5,6,7,8,9,10] or in membranes [11], the formation of networks of endothelial cells [12,13,14,15,16,17,18], and colloidal particles in solution [19,20,21,22,23,24,25] are just some of the most representative examples of SALR systems. In the case of colloids, the driving force for aggregation is associated with an effective short-range attraction, which has an entropic nature and may arise when a depletant is added to a colloidal suspension [26,27,28,29,30]. In fact, such attraction is due to the overlap of colloidal-sphere-excluded volumes, which results in the increase in space available for the smaller-sized depletant species [31], and it may be described by the Asakura–Oosawa theory [32,33]. As depletants, they are usually employed polymers such as hydroxyethylcellulose, polystyrene, and poly(N-isopropylacrylamide) (PNIPAM), or the surfactant sodium dodecyl sulfate (SDS). In turn, colloidal suspensions have been prepared, among others, with silica particles and poly(methyl methacrylate) (PMMA) spheres [20,21,22]. It must be emphasized that the range of the interaction may be controlled by the size of the depletants, i.e., the resulting depletion is shorter ranged when those species are smaller. In addition, the stability of the colloidal suspension depends on several other variables, such as the ionic strength, pH and temperature, that can be experimentally tuned. In addition, specific characteristics of the particles are also important to introduce anisotropy in interparticle interactions, which leads to directional ordering as one observes in the assemblies of patchy particles [34,35].

Charged colloidal particles in the presence of a depletant comprise an SALR system, composed of the abovementioned depletion-induced attraction and a long-range screened electrostatic repulsion. Due to the competition between the attractive and repulsive components, on different length scales, the charged colloidal particles may self-assemble to produce anisotropic structures [36,37,38], even though the interactions are described by isotropic potentials. Indeed, under specific conditions or interaction parameters, the appearance of dense chains of particles, as well as elongated and branched structures, was shown to be possible for SALR systems [7,37,38,39]. Moreover, the experimental evidence of self-assembly of charged colloids [40] opened simple paths to synthesize new materials. More recently, a simple SALR model, obtained by inverse design strategies [41,42,43,44,45,46], was proposed to obtain a great variety of morphologies of clusters resulting from the assembly of spherical colloidal particles [47].

In fact, the strong interest for the assembly of charged colloids has led to an increasing number of studies appearing in the literature over the last decade [7,48,49,50,51,52,53,54,55,56]. In a recent review, Liu and Xi [54] reviewed the parameters of the SALR potentials explored in the literature and proposed a useful classification of the corresponding interactions in three distinct types, according to the magnitude of the range of both the attractive and repulsive components. Thus, the range of the attractive component of both type I and type II potentials is less than 20% of the particle diameter. However, the range of the repulsive part of type I is a fraction of the particle diameter, while it is similar to or even larger than the particle diameter for potentials of type II. In contrast, type III potentials have attractive ranges larger than 20% of the particle diameter, but these are smaller than the respective repulsive components. As shown by Liu and Xi [54], the three types of SALR systems present specific features in their phase diagrams and in the morphology of the clusters. From works in the literature [25,37,38,48,57,58], it appears that interactions of both type I and type II favor the formation of clusters displaying Bernal spirals or analogous motifs. For type II, with a relatively weak (strong) attractive component, however, it is possible to obtain stable (nonequilibrium) clusters, with compact microcrystalline (quasi-linear) structures [48].

In previous works [51,52,53], we employed evolutionary algorithms developed by our group [59,60,61,62] to carry out global optimization calculations on isolated SALR colloidal clusters. The adopted energy model is based on the effective pair-wise potential composed of the Morse function [63] for the attractive part and a Yukawa term to describe the corresponding long-range repulsion. These model functions have parameters that were tuned to explore different interaction ranges; then, by applying the abovementioned criteria for SALR [54], the colloidal systems studied in our works may be classified as type II [51,52] and type III [53]. From the global optimization studies, we have confirmed that low-energy Bernal spirals may be formed for type II potentials, but “beaded-necklace” structures may also arise, and they become the deepest minimum in the larger cluster sizes (i.e., typically around N=19) [51,52]; additionally, ramified clusters have been also discovered [52] for type II potentials. Conversely, the effect of the repulsive term of type III potentials on the structure of the clusters tends to be small (even for large clusters) and, hence, the corresponding lowest-energy minima are preferentially spherical-like motifs [53].

The present work is devoted to the investigation of structural transitions that may occur in type II SALR clusters. Specifically, we have employed our own implementation [64] of the parallel tempering Monte Carlo (PTMC) algorithm [65,66,67,68] to calculate the heat capacity of charged colloidal clusters, which allows the identification of structural transitions occurring at relatively low temperatures. We wonder whether Bernal spirals, “beaded-necklace” and spherical-type structures, that have been assigned as low-energy metastable minima at zero Kelvin [52], can survive at higher temperatures. Besides these structures, it would be interesting to verify whether other motifs are sampled by the present Monte Carlo simulations. On the other hand, it is expected that the dissociation of the cluster can be identified by a strong peak of the heat–capacity curve arising at higher temperatures. The aim of this paper is the characterization of the thermodynamics signature associated with both structural transitions and dissociation of charged colloidal clusters.

## 2. Methods

### 2.1. Interaction Potential

The energy of the cluster has been calculated by assuming a sum over all pair potentials, i.e.,

(1) $$V_{cluster}=\sum_{i=1}^{N(N-1)/2}\left[V_{a}\left(r_{i}\right)+V_{r}\left(r_{i}\right)\right]$$

where $r_{i}$ is the distance between two colloidal particles and the summation runs over all interaction pairs of the cluster. In Equation (1), each pair potential is composed of attractive ($V_{a}$) and repulsive ($V_{r}$) components. It has been shown [69,70] that short-range Morse potentials can reproduce the colloid–colloid depletion attraction, as described by the Asakura–Oosawa theory [32,33]. Accordingly, we have modeled the attractive component of the pair potential by employing the following Morse function [63]:

(2) $$V_{a}\left(r_{i}\right)=\epsilon_{M}\;e^{\;\rho \left(1-r_{i}/\sigma \right)}\;\left\{e^{\;\rho \left(1-r_{i}/\sigma \right)}-2\right\}$$

In Equation (2), $\epsilon_{M}$ denotes the well depth of the Morse potential and σ is the diameter of the colloidal particles, which are assumed to be spheres; the ρ parameter controls the range of the interaction, i.e., higher values of ρ correspond to shorter ranges.

In turn, the repulsive component of the pair potential in Equation (1) can be described by the long-range branch of the Yukawa function, that is,

(3) $$V_{r}\left(r_{i}\right)=\epsilon_{Y}\frac{e^{-\kappa \sigma (r_{i}/\sigma -1)}}{r_{i}/\sigma }$$

We should note that κ is the inverse Debye length and, as in Equation (2), σ designates the diameter of each spherical particle; in addition, $\epsilon_{Y}$ is intimately associated with the charge of the colloidal particles and, hence, determines the strength of the repulsion.

**Figure 1:**
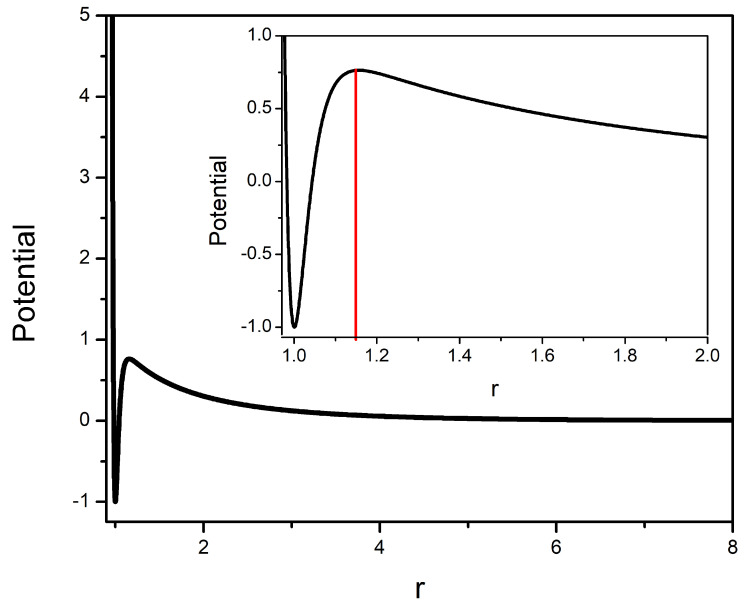
Pair potential model employed for the interaction between two charged colloidal particles. In the insert, the vertical red line indicates the cutoff distance used to define a bound interaction.

In order to allow a fair confrontation of the present results with those from our previous global optimization studies on colloidal clusters [51,52], we have used here the same values for the abovementioned potential parameters. In reduced units, the values of the parameters are: ρ=30, $\epsilon_{M}=2.0$, σ=1, κσ=0.5 and $\epsilon_{Y}=1.0$. It is worth noting that such parameters correspond to the potential model designated as M30+Y1.0 in Refs. [51,52]. The M30+Y1.0 pair potential is displayed in Figure 1. As can be observed, this is a typical type II SALR potential, displaying a short-range well and a barrier with a maximum at r≈1.15.

### 2.2. Parallel Tempering Monte Carlo Method

The parallel tempering method is employed to explore the energy landscape and dissociation thermodynamics of colloidal clusters. The present implementation follows, in general, the previous one discussed in Ref. [64], where we studied the microsolvation of ions by rare gas. Thus, we highlight here the small differences due to the characteristics of the interaction potentials used to model the title systems.

Within the canonical ensemble, where the number of particles (*N*), the temperature (*T*) and the volume (*V*) are constants, the heat capacity per particle, or reduced heat capacity, is given by the following expression:

(4) $$c_{V}=\frac{3}{2}k+\frac{\langle V_{cluster}^{2}\rangle -{\langle V_{cluster}\rangle }^{2}}{N{\left(kT\right)}^{2}}\;,$$

where *k* is the Boltzmann constant.

The average terms $\langle V_{cluster}\rangle$ and $\langle V_{cluster}^{2}\rangle$ in Equation (4), determined from the 3N-dimensional integrals, can be numerically evaluated through a Monte Carlo simulation by using the Metropolis algorithm:

(5) $$\langle V_{cluster}^{q}\rangle \approx \frac{1}{N_{steps}}\sum_{i}^{N_{steps}}{\left[V_{cluster}\left(R_{i}\right)\right]}^{q}\;,\;\;q=1,2\;,$$

where $\left\{R_{i}\right\},i=1,\cdots ,N_{steps},$ is a random walk generated according to the following probability distribution:

(6) $$\rho \left(R_{i}\right)=\frac{e^{\left[-V_{cluster}\left(R_{i}\right)/kT\right]}}{Z}\;.$$

In such a case, *Z* is the configurational integral and R represents a 3N-dimensional vector with the position of the *N* particles of the system.

The PTMC strategy consists is the construction of an ensemble with *M* different independent simulations (using the Metropolis algorithm), each of them is in thermal contact with a reservoir at a distinct temperature, $T_{m}$. These simulations are performed in parallel, and attempts to swap configurations at different temperatures are made after a certain number of MC steps, according to the following acceptance probability:

(7) $$A\left(R_{i}^{\left(m\right)}\to R_{i}^{\left(p\right)}\right)=\min\left\{1,e^{\left[\left(V_{cluster}\left(R_{i}^{\left(m\right)}\right)-V_{cluster}\left(R_{i}^{\left(p\right)}\right)\right)\left(1/kT_{m}-1/kT_{p}\right)\right]}\right\}\;,$$

where $R_{i}^{\left(m\right)}$ and $R_{i}^{\left(p\right)}$ are the *i*-th step of the Metropolis random walk associated with temperatures $T_{m}$ and $T_{p}$, respectively. The success of the method mainly depends on the algorithms used to exchange the replicas.

The potential model employed in this work presents an energy barrier that favors dissociation when the particles move away more than the cutoff distance, $r_{c}$, indicated by the red vertical line in Figure 1. Thus, some details need to be introduced in the present implementation, which we describe next.

First, the initial configuration $\left(R_{1}\right)$ in the Metropolis algorithm will be always the global minimum structure reported in Ref. [52], with a thermalization stage prior to the production one. This avoids the appearance of fragmented subclusters at the beginning of the simulation, which would occur with a random generation of the initial configurations.

Another problem that arises in charged colloidal systems is the fragmentation of the system when a particle (or a sub-aggregate), during an MC step, is placed at a distance greater than $r_{c}$ (i.e., beyond the red line in Figure 1). One way to avoid this phenomenon is to introduce another criterion for the acceptance of a Monte Carlo step, so that any proposed movement of a particle causing the cluster rupture is not allowed. That is, if in the proposed new configuration, at least one particle is at a distance greater than the cutoff $r_{c}$ from all the others, then the MC step is not accepted, and R remains unchanged.

Other crucial aspects in PTMC are the choice of the $\left\{T_{m}\right\}$ temperature set to be used by each independent simulation, the exchange process of structures between different temperature simulations, and the maximum size of the MC step. For the former, we have chosen $\left\{T_{m}\right\}$ from constant steps in predefined intervals. This procedure was used by us in a previous work when treating larger Li${}^{+}$Ar${}_{n}$ clusters [64]. To perform the exchange of configurations between Metropolis simulations at different temperatures, we have employed the efficient deterministic even–odd (DEO) algorithm [71,72]. Moreover, we have used the step-sized adjustment proposed in Ref. [73] to maintain an acceptance rate of 50% for the MC moves carried out at each temperature in the parallel tempering strategy.

In order to identify the main structures arising in each simulation, we have stored thousands of non-correlated configurations for all considered temperatures during the PTMC calculations. Then, local optimizations of each one of these configurations were performed, which is thus relaxed to a stable structure within the same energy landscape basin. Relaxed structures with energies differing by less than 1 ×$10^{-6}$ (in reduced units) were considered as being equal.

## 3. Results and Discussion

The thermodynamics signature of structural transitions arising in charged colloidal clusters may be apparent in the curve of the heat capacity calculated at constant volume. Accordingly, the parallel tempering Monte Carlo calculations carried out in the present work allowed us to display the heat–capacity curve from very low temperatures to those slightly beyond the cluster dissociation. It is in such temperature range that structural transitions may be observed for a given cluster. To facilitate the presentation of the results, we have separated the analysis in two subsections: one for the smaller clusters (i.e., from N=9 up to N=12), while the other covers larger sizes (i.e., from N=17 up to N=20). Indeed, our previous global optimization study on the same charged colloidal clusters has shown significant qualitative changes in the structure of the global minimum at these size ranges [52].

The $c_{V}$ results presented in this section were obtained from 3 independent PTMC runs with $N_{steps}=15\times 10^{9}$ after a thermalization stage of $5\times 10^{9}$ steps; the temperature exchange procedure is performed every 50 MC steps. We emphasize that such a great number of overall steps is required to achieve a reasonable convergence of the $c_{V}$ values for these colloidal clusters. Furthermore, M=26 temperatures in the range of 0.05–0.30 (with ΔT=0.1) were considered for all clusters, with the exception of the one with 11 particles (i.e., N=11). For this case, M=33 temperatures were considered in the same range, but we have rather employed ΔT=0.05 in the interval between T=0.11 and T=0.18. Moreover, this has shown to be particularly relevant for improving the convergence of the calculation around the first peak for N=11 (see below).

Within the described conditions for PTMC calculations, the error in $c_{V}$ has been estimated by computing the standard deviation from the three independent PTMC runs at each temperature. In general, these errors are smaller than 1%, while higher values (∼6%) are obtained in a limited number of temperatures around the predissociation peaks (see discussion in Section 3.1). Thus, such errors are sufficiently small to extract unambiguous information from the Monte Carlo calculations.

### 3.1. Clusters with N = 9–12 Particles

Charged colloidal clusters in the size range N=9–12 show [51,52] Bernal spirals for the global minima of N=9 and 11, while the corresponding ones for N=10 and 12 are spherical-type structures. Thus, it is likely to expect a competition between Bernal spirals and spherical-type shapes for this cluster size range. Moreover, we want to investigate whether these two types of structures are thermodynamically stable and the transition between them is likely to occur at a given temperature prior to dissociation.

**Figure 2:**
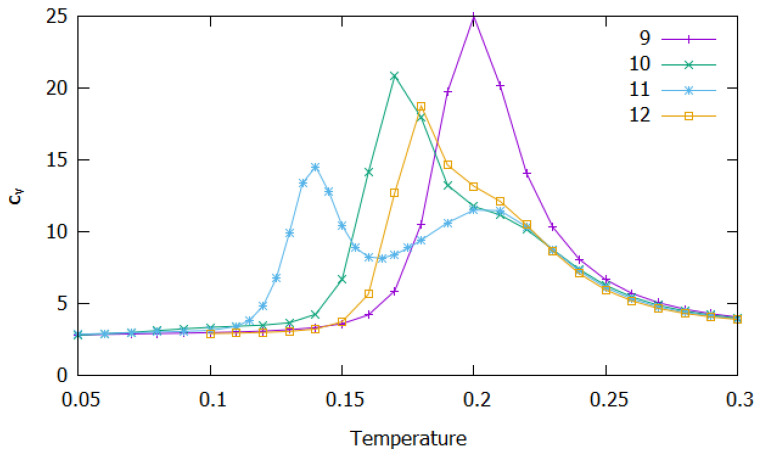
Reduced heat capacity as a function of temperature (in reduced units) for small cluster sizes: N=9 (magenta); N=10 (green); N=11 (blue); N=12 (orange).

In Figure 2, we display the reduced heat capacity at constant volume ($c_{V}$) as a function of temperature for N=9–12. A first glance at Figure 2 shows that a single peak arises in the $c_{V}$ curves of N=9,10 and 12. Nonetheless, the $c_{V}$ curves for N=10 and N=12 display a small shoulder that broadens the prominent peak towards temperatures around 0.20. By contrast, two peaks are shown for N=11. As we will explain below, the peak or shoulder arising at higher temperatures can be ascribed to the dissociation of the cluster.

**Figure 3:**
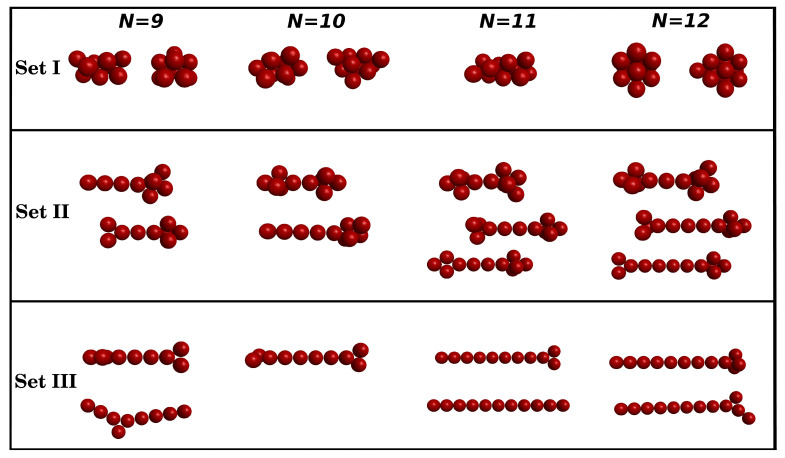
Main structural motifs from Set I, Set II and Set III of smaller colloidal clusters (N=9–12); see the main text.

Since the specific features of the $c_{V}$ curves may be related to structural transformations occurring in the clusters as a function of temperature, we have been monitoring the structures that were formed during the PTMC calculation for each cluster size. From all the relaxed structures obtained in the local optimization stage (as described in end of Section 2), we have considered as main structural motifs those that arise with a frequency greater or equal than 4% for at least one temperature. In Figure 3, we represent some of the main structural motifs that were found; for completeness, all the main structures and corresponding energies are reported in the Supplementary Materials. Such structures are separated in three sets (*cf.* Figure 3) that are characterized as follows. Set I contains low-energy structures (including the global minima discovered in Ref. [52]), which are either spherical-type shapes or Bernal spirals; for N=9,10 and 12, the left-side structure is the global minimum, while the ones on the right side show small deformations of such compact shapes. In turn, “beaded-necklace” structures arise in Set II, whereas Set III contains branched and linear motifs. We should emphasize that “beaded-necklace” and other linear motifs are increasingly higher energy structures (as it can be seen in the Supplementary Materials), which may result from unrolling the Bernal spiral; note especially that structures from Set II still keep a little part of the 3D shape and, by contrast, the motifs in Set III are essentially unrolled.

**Figure 4:**
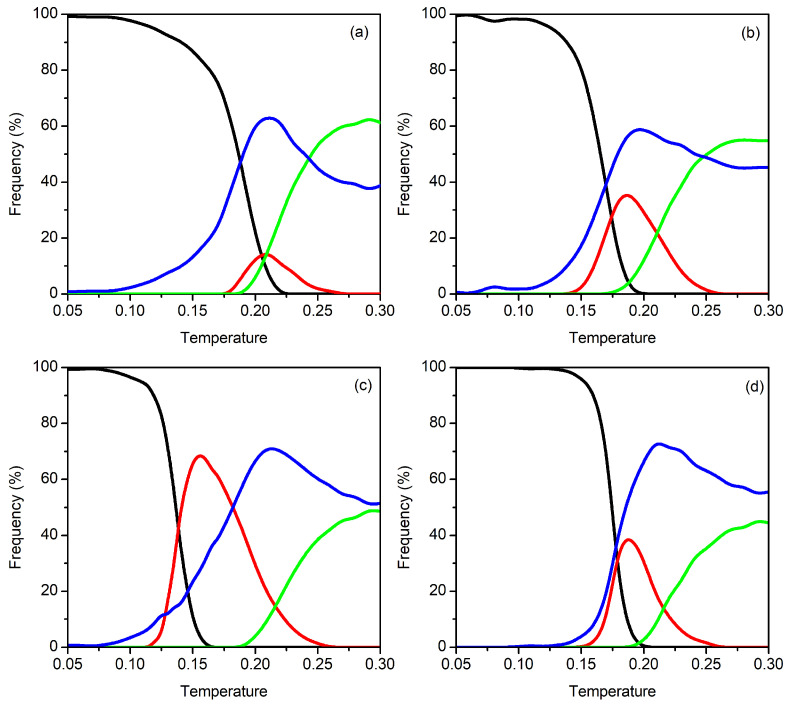
Frequency of the structural motifs as a function of temperature: (**a**) N=9; (**b**) N=10; (**c**) N=11; (**d**) N=12. Besides low-frequency miscellaneous motifs (blue), lines corresponding to other sets of structures, as defined in Figure 3, are also indicated: Set I (black); Set II (red); Set III (green). At a given temperature, 100% frequency is obtained when summing up the corresponding values for Set I, Set II, Set III and miscellaneous structures.

As an attempt to explain the features of the $c_{V}$ curves described above, in Figure 4, we show the frequency of the main structural motifs (Set I, Set II and Set III) as a function of temperature for N=9–12. Additionally, the blue line in Figure 4 represents the cumulative contribution of all the structures whose individual frequency is always below 4%. These are designated, henceforward, as “miscellaneous structures”, and they correspond to a panoply of minima from the energy landscape that, though with small probability, may become accessible with increasing temperature. Indeed, the number of such miscellaneous structures can reach hundreds at high temperatures, which is an indication of the great structural diversity associated with very narrow minima that are spread across the energy landscape. This is also compatible with a certain fluidity of the cluster that usually occurs slightly below the dissociation temperature [64,74]. We emphasize that, due to the number of miscellaneous structures found, we did not classify them in terms of Sets I, II and III, and, thus, they may have different structural characteristics.

A first glance at Figure 4 shows that, regardless the cluster size, structures from Set I dominate at low temperatures. In turn, the “beaded-necklace” (Set II) and the branched and linear (Set III) structures abundantly arise in the PTMC calculations at higher temperatures. We should emphasize that the formation of separated subclusters is prevented in the present calculations (see Section 2) and, hence, the appearance of a great number of linear structures from Set III (green curve) for temperatures above the $c_{V}$ peak, is clearly an indication of the formation of a dissociated state.

Moreover, we observe in Figure 4 that, as the temperature increases, structures from Set I tend to decrease their frequency, while other type of structures gain relevance. For N=9, the frequency of structures from Set I decrease at slow rate up to *T*∼0.17, whereas it decays rapidly for higher temperatures. In the same range of temperature, an increase is shown in the collective frequency of the abovementioned miscellaneous structures (blue curve), which is due to the appearance of a great number of clusters with different motifs (each one with low frequency). This competition between structures of Set I and miscellaneous motifs characterizes a predissociative fluidity of the cluster and, hence, explains the $c_{V}$ peak arising at T=0.20, which may be ascribed to dissociation. Slightly before this temperature, “beaded-necklace” (Set II) and linear or branched structures (Set III) begin to appear with a reasonable frequency. The reason for the appearance of structures from Set II only at high temperatures might be due to the energy gap between Set I and Set II, as shown in Table S1 of Supplementary Materials. In turn, similar features are observed for the frequency of the different sets of structures as a function of temperature in the cases of N=10 and N=12. For these cluster sizes, however, the fast decay of the frequency of structures from Set I and the appearance of motifs from Set II, slightly before dissociation (which occurs at *T*∼0.20 as shown by the green curve of Set III structures in Figure 2) leads to an overlap between two expected $c_{V}$ peaks. The first of these peaks is ascribed to the structural transition between Set I and Set II and arises at T=0.17 (T=0.18) for N=10 (N=12), while the second one corresponds to the dissociation of the cluster and is perceived by the shoulder at *T*∼0.20. Moreover, the first peak displayed by the $c_{V}$ curves immediately before the dissociation temperature can be explained based on the greater importance assumed by “beaded-necklace” structures (red curve, with a maximum exceeding 30%). By contrast, the maximum of the red curve for N=9 coincides with the dissociation peak, which becomes the highest one when compared with the other cluster sizes.

Conversely, it is shown in Figure 4 that, for N=11, there is an earlier competition between structures from Set I and Set II. Actually, the decay of the frequency of structures from Set I occurs at low temperatures, i.e., the corresponding curve fall off to zero at around T=0.15, while “beaded-necklace” motifs become preponderant by reaching a maximum frequency of ∼75%. It is worth noting that the collective frequency of miscellaneous structures is, now, smaller (i.e., reaching only about 25%) than the “beaded-necklace” ones at the referred temperature range. Clearly, this constitutes a structural transition between the Bernal spiral (Set I) and the “beaded-necklace” motifs (Set II), whose signature arise as a low-temperature peak in the $c_{V}$ curve of N=11 (*cf.* Figure 2). This happens well below dissociation, which also occurs at T=0.20 and is accompanied by the enhancement of miscellaneous motifs (blue curve) at the expenses of those from Set II. Furthermore, linear and branched motifs (green curve) become significant for T≥0.20, which is a clear indication of a dissociative state.

### 3.2. Clusters with N = 17–20 Particles

**Figure 5:**
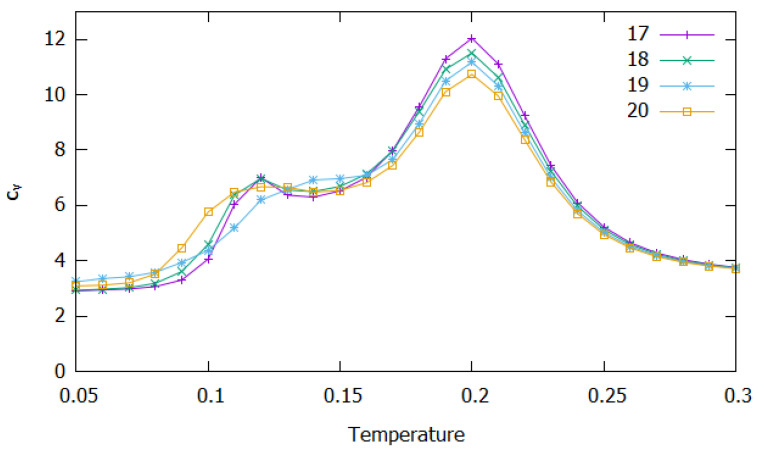
Reduced heat capacity as a function of temperature (in reduced units) for the cluster sizes of N=17 (magenta), N=18 (green), N=19 (blue), and N=20 (orange).

**Figure 6:**
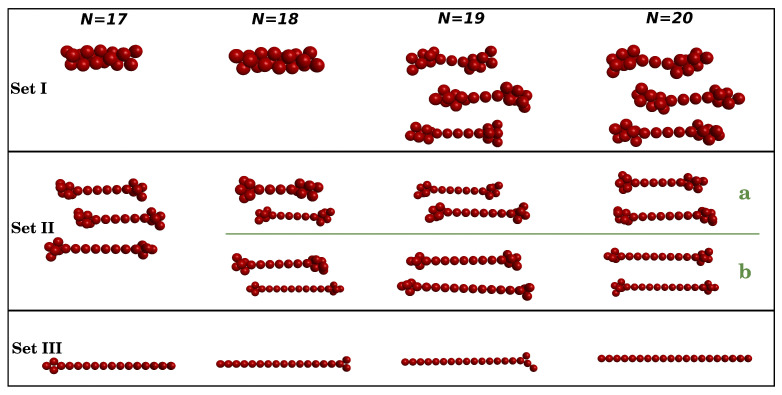
Main structural motifs from Set I, Set II and Set III of larger colloidal clusters (N=17–20). For N=18–20, structures from Set II are separated into Set IIa and Set IIb (the latter are higher energy than the former ones). See the text for details.

The calculated heat capacity as a function of reduced temperature is displayed in Figure 5 for the cluster sizes N=17,18,19 and 20. A first glance at this figure immediately shows that the highest peak arises at about T=0.20 for all clusters; similarly to the lower-size clusters, this peak can be ascribed to dissociation (see also below). Thus, it appears that the dissociation temperature is independent of the size of the clusters. Nonetheless, the value of $c_{V}$ at the maximum tends to diminish with the increasing size of these large clusters, which may be understood if we have in mind that SALR potentials lead to less-bounded structures as *N* increases [51,52]. Note in particular that the lowest-energy structures for these larger clusters (Set I in Figure 6) are already Bernal spirals (N=17 and 18) or “beaded-necklace” motifs (N=19 and 20) and the corresponding values of energy per particle are −1.423, −1.395, −1.376 and −1.364 (see Supplementary Materials and Table II of Ref. [52]), i.e., the contribution of each particle to the stability of the cluster decreases as *N* increases.

As was performed for the smaller clusters, we have separated the main structural motifs into three sets in order to understand other important features that appear in the $c_{V}$ curves of Figure 5. However, Set I of N=17 and 18 contains only the global minimum structure (Bernal spirals), while the clusters with N=19 and N=20 present a significant number of different “beaded-necklace” motifs (12 and 11 structures, respectively) distributed in a smaller range of energy (see Table S1). Additionally, Set II of N=18, 19 and 20 were divided into two subsets, identified as Sets IIa and IIb, which can contribute to understand some features of the $c_{V}$ curves in the predissociation region. Despite being a somehow arbitrary separation, Set IIb contains more elongated “beaded-necklace” motifs, with their terminations having fewer particles (in comparison with those in Set IIa), as can be seen from the examples displayed in Figure 6. Furthermore, we note that the energy difference between structures from Set III and Set II for N=17–20 is much larger than that observed for the smaller cluster sizes in Section 3.1 (see also Table S1).

**Figure 7:**
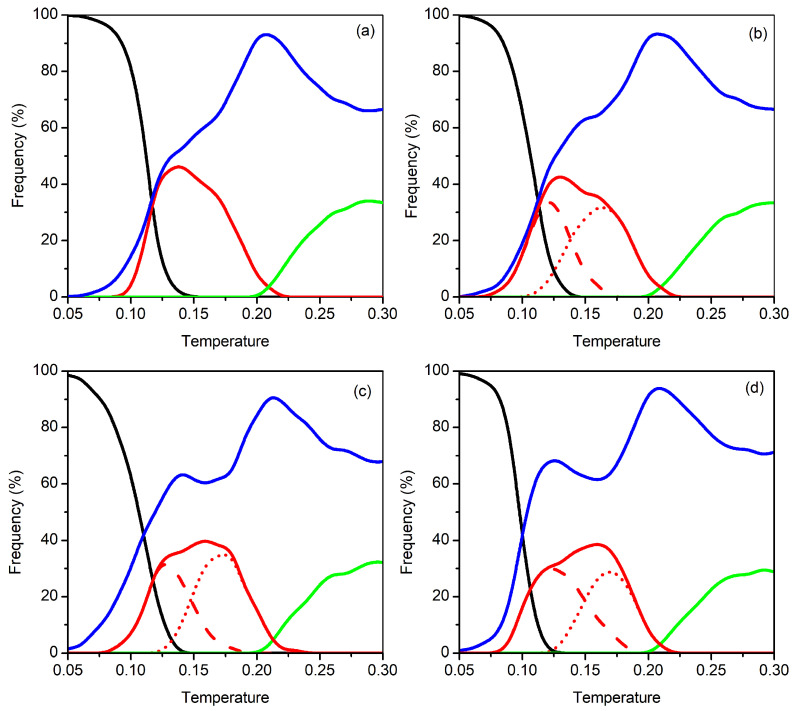
Frequency of the structural motifs as a function of temperature: (**a**) N=17; (**b**) N=18; (**c**) N=19; (**d**) N=20. Besides low-frequency miscellaneous motifs (blue), lines corresponding to other sets of structures, as defined in Figure 6, are also indicated: Set I (black); Set II (red); Set III (green). For N=18−20, red dashed and red dotted lines correspond, respectively, to structures from Set IIa and Set IIb (see Figure 6).

We can also observe in Figure 5 that other significant feature arise in the $c_{V}$ curves for temperatures below dissociation. Specifically, a small maximum is apparent at T=0.12 for N=17,18 and 20, while a shoulder that extends up to T=0.16 appears in the case of N=19. This predissociation feature may be rationalized by the inspection of Figure 7 that represents the frequency of the different structural motifs shown in Figure 6 as a function of temperature. It is apparent from Figure 7 that the frequency of structures from Set I decays very rapidly with temperature in all cases. Indeed, structures from Set I are absent for T>0.15 and, by contrast, structures from Set II (red lines) and miscellaneous motifs (blue lines) begin to appear at low temperatures (for T<0.10). This somehow resembles the behavior described above for N=11 [*cf.* Figure 4c], which also displays a predissociation $c_{V}$ peak due to a structural transition. However, such peak is stronger for N=11 than in the case of the larger sizes, since the frequency of structures from Set II (red curve) becomes much higher for that smaller cluster. Actually, as pointed out in Section 3.1, the frequency of structures from Set II for N=11 dominates over all other types of structures in a limited range of temperature; specifically, the red curve in Figure 4c overcomes all other curves and, in particular, the blue one for the miscellaneous structures that are expected to arise at the expenses of either Bernal spiral or “beaded-necklace”. Conversely, such a dominance of structures from Set II over other type of structures is not observed, now, for the larger clusters.

Accordingly, we may justify the appearance of the small $c_{V}$ peak at T=0.12 for N=17 and 18 by the high frequency of structures from Set II, which is similar but, nonetheless, does not overcome the one for miscellaneous motifs; hence, this $c_{V}$ peak may be ascribed to a structural transition between the Bernal spirals and the motifs from Set II, which appears to benefit from a certain predissociation fluidity of the cluster (shown by the high frequency of miscellaneous structures). By contrast, we observe in Figure 7 that, for N=19 and N=20, the frequency of structures from Set II is always lower than that for the miscellaneous motifs; such difference is greater for N=19. Thus, this explains the shoulder observed in the $c_{V}$ curve for N=19, whereas a very small and broad maximum arises at predissociation temperatures for N=20 (see Figure 5). Moreover, we should also note that the shoulder for N=19 is slightly displaced to higher temperatures (*T*∼0.15) in relation to the predissociation peak arising at *T*∼0.12 for N=17,18 and 20. By the inspection of Figure 7, we conclude that such shoulder might be attributed to a transition between structures from Set IIa (red dashed curve) and Set IIb (red dotted curve). Indeed, the dotted curve is slightly higher than the dashed one for N=19, whereas both red lines present essentially the same height for N=18 and N=20.

## 4. Conclusions

We have performed parallel tempering Monte Carlo calculations for charged colloidal systems in order to study the thermodynamics of the cluster dissociation and to assess predissociation structural transitions. The potential model is based on effective interactions involving pairs of colloidal particles, which is composed of a short-range attraction described by a Morse function and a long-range repulsion represented as an Yukawa form. To our knowledge, this is the first Monte Carlo study involving charged colloidal clusters. Actually, as we have confirmed in the present work, the convergence of the PTMC calculations requires more than $10^{9}$ thermalization steps (and even more for some specific cluster sizes), which may be attributed to the energy landscape that is expected to show very narrow minima connected by high barriers.

The present Monte Carlo simulations show that the low-energy clusters may be spherical-type (for N=9,10, and 12), Bernal spirals (for N=9,11,17, and 18) or “beaded-necklace” (for N=19 and 20) structures, which is in agreement with our global optimization study [51,52] performed on these systems. Other sets of structures having higher energy, essentially resulting from unrolling the Bernal spiral, arise as the temperature increases. For some sizes (N=11,17, and 18), such structures become significant prior to dissociation leading to a peak of the $c_{V}$ curve at intermediate temperatures. In particular, a strong peak appears at T=0.12 for N=11, which constitutes a clear signature of the structural transition. In turn, the dissociation occurs for all studied clusters at around T=0.20, while the temperature at which the structural transition between Set I and Set II motifs arises depends on the specific cluster. In addition, the $c_{V}$ signature of the predissociation structural transition may arise as a peak (for N=10–12 and N=17,18 and 20) or as a shoulder (for N=19). In the case of N=9, such structural transition coincides with the dissociation and, hence, it shows only one enhanced peak at T=0.20. Additionally, it is important to emphasize that the clusters for N=11,17,18 and 20 show a clear separation between the structural transition and the dissociation. Conversely, there is no such clear separation for all the other studied clusters. Specifically, the structural transition shows a prominent $c_{V}$ peak for N=10 and N=12 that somehow obscures the dissociation of the clusters. For N=19, by contrast, the structural transition is less marked and, now, it is the dissociation that presents a significant peak in the $c_{V}$ curve.

Finally, we should emphasize that the parameter $\epsilon_{Y}$ of the repulsive Yukawa term is assumed to be constant, i.e., the long-range repulsion is proportional to the charge of the colloidal particles. Nonetheless, it has been shown [52] that the shape of the global minimum and other minima vary for different values of $\epsilon_{Y}$. Thus, such assumption may have influence on the calculated $c_{V}$ as a function of temperature. In a future work, we will study the effect of $\epsilon_{Y}$ on the features of the $c_{V}$ curve.

## Data Availability

Data are contained within the article or supplementary material.

## Supplementary Materials

The following supporting information can be downloaded at: https://www.mdpi.com/article/10.3390/molecules27082581/s1. Table S1. Energy range of the structures from Set I, Set II, and Set III for each cluster size.
