# Supplementary material for: Thermodynamic Signatures of Structural Transitions and Dissociation of Charged Colloidal Clusters: A Parallel Tempering Monte Carlo Study
Source: Molecules. 2022 Apr 16;27(8):2581. doi: 10.3390/molecules27082581 (PMC9032479; doi:10.3390/molecules27082581)
Supplement: Supplementary file 1 [file molecules-27-02581-s001.zip › molecules-1644630-supplementary.pdf]

# Thermodynamics signature of structural transitions and dissociation of charged colloidal clusters: a parallel tempering Monte Carlo study

Frederico V. Prudente[1] and Jorge M. C. Marques[2]

[1] *Instituto de Física, Universidade Federal da Bahia, 40170-115 Salvador, BA, Brazil; prudente@ufba.br*

[2] *CQC, Department of Chemistry, University of Coimbra, 3004-535 Coimbra, Portugal; qtmarque@ci.uc.pt*

**Table S1.** Energy range of the structures from Set I, Set II, and Set III for each cluster size. For the Set I of $N$=17 and $N$=18, there is only one structure whose energy is indicated.

| $N$ | **Set I** | **Set II** | **Set III** |
|---|---|---|---|
| 9 | (−15.562,−15.290) | (−10.790,−9.372) | (−7.558,−4.702) |
| 10 | (−17.211,−16.581) | (−13.885,−9.810) | (−8.922,−5.168) |
| 11 | (−18.091,−17.748) | (−15.516,−10.261) | (−8.472,−5.632) |
| 12 | (−19.338,−18.580) | (−16.356,−10.717) | (−8.933,−6.096) |
| 17 | −24.198 | (−23.181,−17.634) | (−10.183,−8.414) |
| 18 | −25.108 | a: (−24.538,−22.779) | (−10.646,−8.877) |
| | | b: (−21.826,−18.099) | |
| 19 | (−26.154,−24.902) | a: (−24.332,−22.304) | (−11.109,−9.304) |
| | | b:(−21.208,−16.785) | |
| 20 | (−27.291,−26.296) | a: (−25.773,−22.774) | (−11.572,−9.804) |
| | | b: (−21.657,−19.027) | |

**Small clusters data**

*N*=9

SET I:

| | | |
|---|---|---|
| 9A<br>Energy: -15.56156020<br>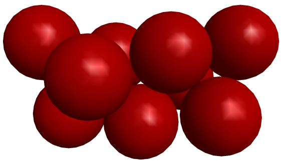 | 9B<br>Energy: -15.55806630<br>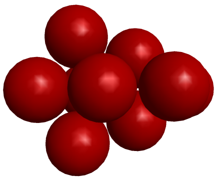 | 9C<br>Energy: -15.46152621<br>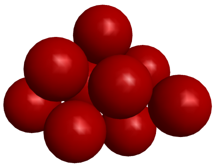 |
| 9D<br>Energy: -15.37419913<br>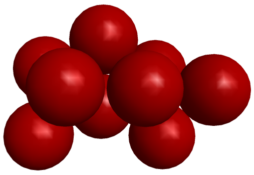 | 9E<br>Energy: -15.35417435<br>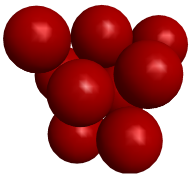 | 9F<br>Energy: -15.28992983<br>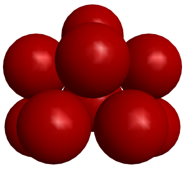 |

SET II

| | | |
|---|---|---|
| 9G<br>Energy: -10.78993014<br>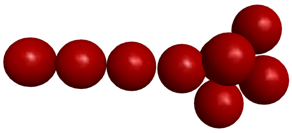 | 9H<br>Energy: -10.21788243<br>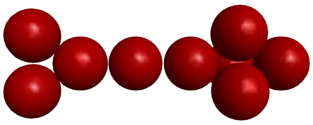 | 9I<br>Energy: -9.37254143<br>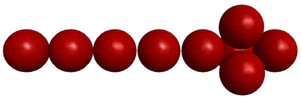 |

SET III

| | | |
|---|---|---|
| 9J<br>Energy: -7.55783121<br>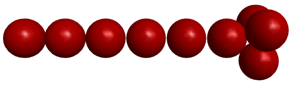 | 9K<br>Energy: -7.39310189<br>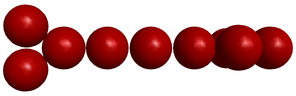 | 9L<br>Energy: -6.54710392<br>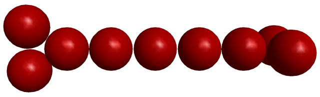 |

| | | |
|---|---|---|
| 9M<br>Energy: -6.48545675<br>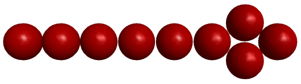 | 9N<br>Energy: -5.62736246<br>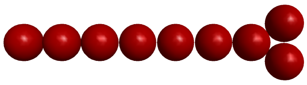 | 9O<br>Energy: -5.24671446<br>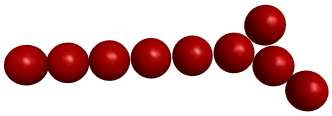 |
| 9P<br>Energy: -5.10267896<br>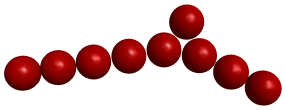 | 9Q<br>Energy: -4.70201501<br>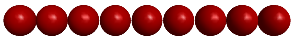 | |

*N*=10

SET I

| | | |
|---|---|---|
| 10A<br>Energy: -17.21101095<br>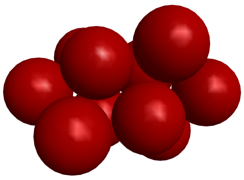 | 10B<br>Energy: -16.87425093<br>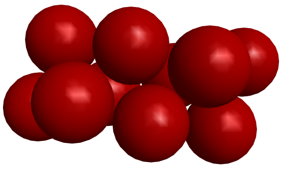 | 10C<br>Energy: -16.68045730<br>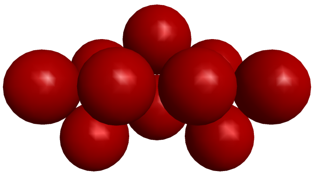 |
| 10D<br>Energy: -16.64222794<br>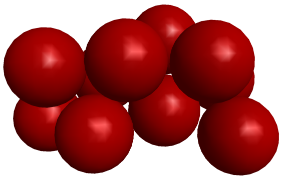 | 10E<br>Energy: -16.62190950<br>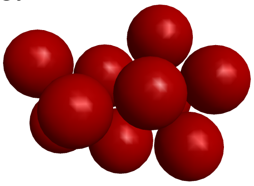 | 10F<br>Energy: -16.58118497<br>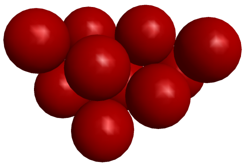 |

SET II

| | | |
|---|---|---|
| 10G<br>Energy: -13.88520948<br>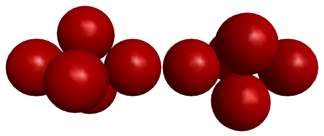 | 10H<br>Energy: -12.46579791<br>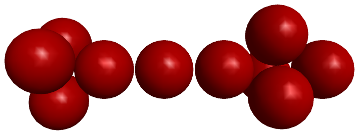 | 10I<br>Energy: -12.00287046<br>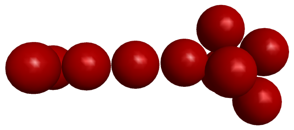 |
| 10J<br>Energy: -11.46719574<br>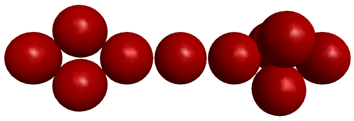 | 10K<br>Energy: -11.18262418<br>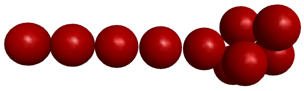 | 10L<br>Energy: -10.69663547<br>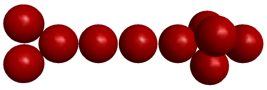 |
| 10M<br>Energy: -9.81047846<br>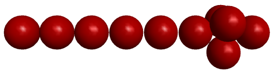 | | |

SET III

| | | |
|---|---|---|
| 10N<br>Energy: -8.92247264<br>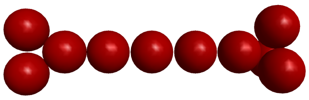 | 10O<br>Energy: -8.01291959<br>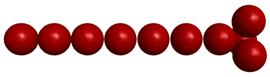 | 10P<br>Energy: -7.85884213<br>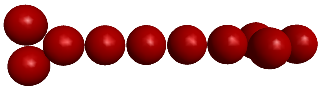 |
| 10Q<br>Energy: -7.01105861<br>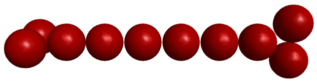 | 10R<br>Energy: -6.94437832<br>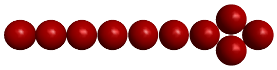 | 10S<br>Energy: -6.09081450<br>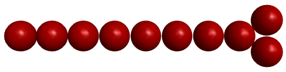 |

| | | |
|---|---|---|
| 10T<br>Energy: -5.70562160<br>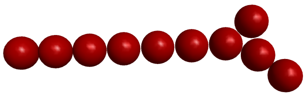 | 10U<br>Energy: -5.16777479<br>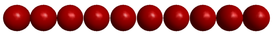 | |

*N*=11

SET I

| | | |
|---|---|---|
| 11A<br>Energy: -18.09068047<br>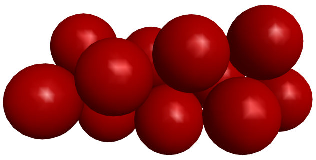 | 11B<br>Energy: -17.85065043<br>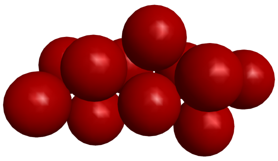 | 11C<br>Energy: -17.80311256<br>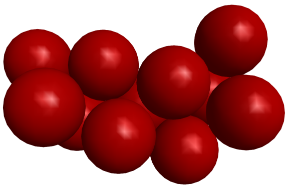 |
| 11D<br>Energy: -17.74788989<br>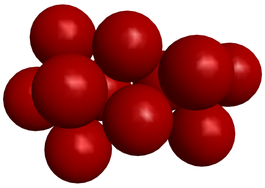 | | |

SET II

| | | |
|---|---|---|
| 11E<br>Energy: -15.51605893<br>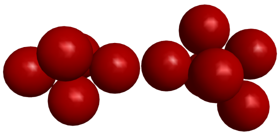 | 11F<br>Energy: -14.78362454<br>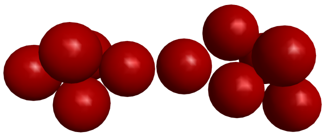 | 11G<br>Energy: -14.64097653<br>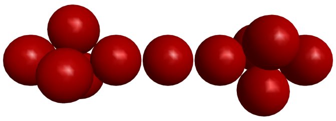 |

11H
Energy: -14.20555101

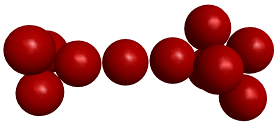

11I
Energy: -13.57236423

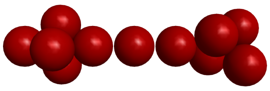

11J
Energy: -13.01227628

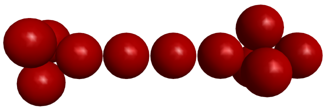

11K
Energy: -12.48451081

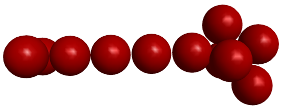

11L
Energy: -11.97787636

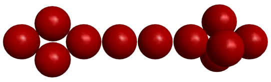

11M
Energy: -11.16475660

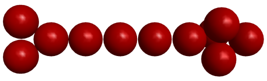

11N
Energy: -10.26084011

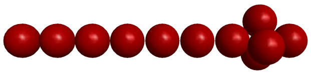

SET III

11O
Energy: -8.47198864

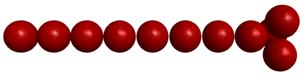

11P
Energy: -7.40544575

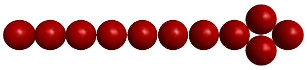

11Q
Energy: -6.55424225

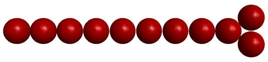

11R
Energy: -6.16667287

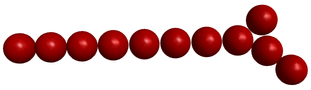

11S
Energy: -5.63242993

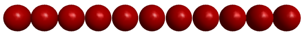

*N*=12

SET I

| | | |
|---|---|---|
| 12A<br>Energy: -19.33769398<br>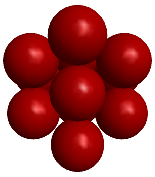 | 12B<br>Energy: -18.58018841<br>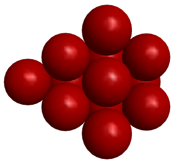 | |

SET II

| | | |
|---|---|---|
| 12C<br>Energy: -16.35607159<br>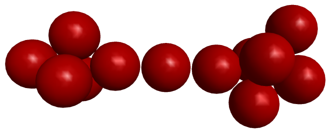 | 12D<br>Energy: -15.51831926<br>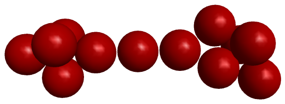 | 12E<br>Energy: -15.22193037<br>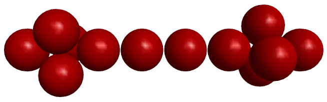 |
| 12F<br>Energy: -14.77758078<br>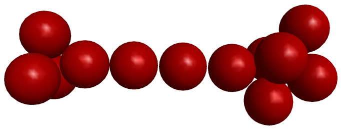 | 12G<br>Energy: -14.12146597<br>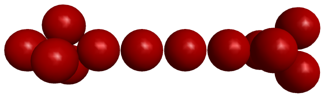 | 12H<br>Energy: -13.51098250<br>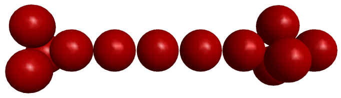 |
| 12I<br>Energy: -12.46106057<br>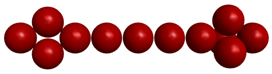 | 12J<br>Energy: -11.62952326<br>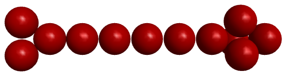 | 12K<br>Energy: -10.71737568<br>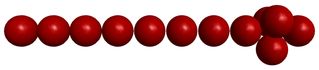 |

SET III

| | | |
|---|---|---|
| 12L<br>Energy: -8.93305764<br>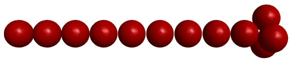 | 12M<br>Energy: -7.86757644<br>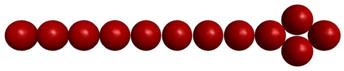 | 12N<br>Energy: -7.01762623<br>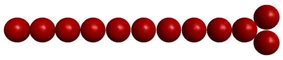 |
| 12O<br>Energy: -6.62879110<br>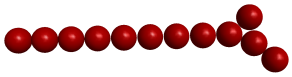 | 12P<br>Energy: -6.09647630<br>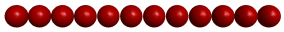 | |

**Larger clusters data**

*N*=17

SET I

| | | |
|---|---|---|
| 17A<br>Energy: -24.19775922<br>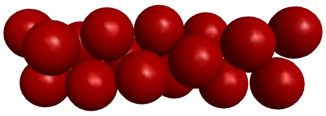 | | |

SET II

| | | |
|---|---|---|
| 17B<br>Energy: -23.18147845<br>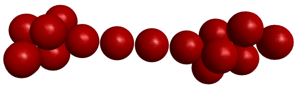 | 17C<br>Energy: -22.36142585<br>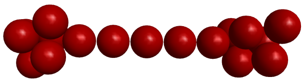 | 17D<br>Energy: -22.36142245<br>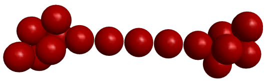 |
| 17E<br>Energy: -22.25523432<br>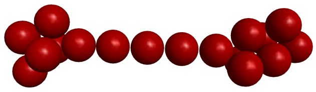 | 17F<br>Energy: -21.33582304<br>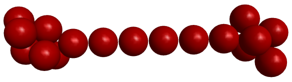 | 17G<br>Energy: -20.26432848<br>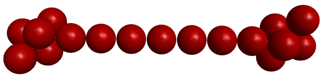 |
| 17H<br>Energy: -20.04639508<br>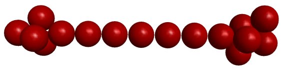 | 17I<br>Energy: -18.95418110<br>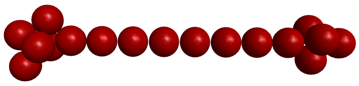 | 17J<br>Energy: -17.63445734<br>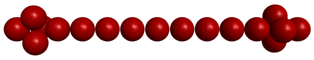 |

SET III

| | | |
|---|---|---|
| 17K<br>Energy: -10.18269373<br>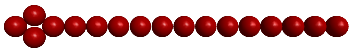 | 17L<br>Energy: -9.33416592<br>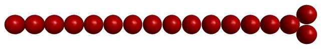 | 17M<br>Energy: -8.94388763<br>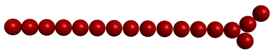 |
| 17N<br>Energy: -8.41378484<br>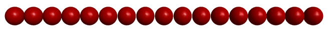 | | |

*N*=18

SET I

| | | |
|---|---|---|
| 18A<br>Energy: -25.10845390<br>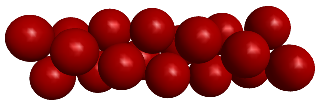 | | |

SET IIa

| | | |
|---|---|---|
| 18B<br>Energy: -24.53813820<br>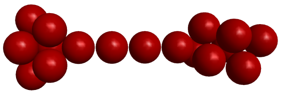 | 18C<br>Energy: -23.79528244<br>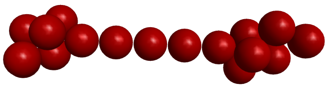 | 18D<br>Energy: -23.79528024<br>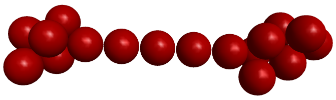 |
| 18E<br>Energy: -23.75460477<br>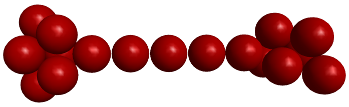 | 18F<br>Energy: -22.89050733<br>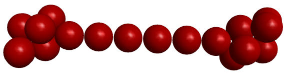 | 18G<br>Energy: -22.89050611<br>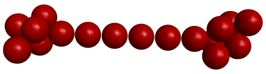 |
| 18H<br>Energy: -22.77932744<br>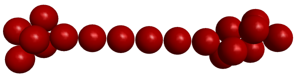 | | |

SET IIb

| | | |
|---|---|---|
| 18I<br>Energy: -21.82630174<br>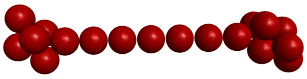 | 18J<br>Energy: -20.73902524<br>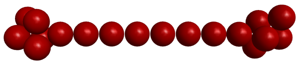 | 18K<br>Energy: -20.51963552<br>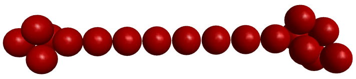 |
| 18L<br>Energy: -19.42168115<br>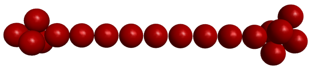 | 18M<br>Energy: -18.09930357<br>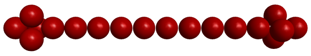 | |

SET III

| | | |
|---|---|---|
| 18N<br>Energy: -10.64593661<br>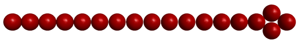 | 18O<br>Energy: -9.79744424<br>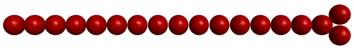 | 18P<br>Energy: -9.40712976<br>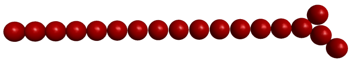 |
| 18Q<br>Energy: -8.87708291<br>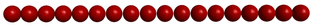 | | |

*N*=19

SET I

| | | |
|---|---|---|
| 19A<br>Energy: -26.15384179<br>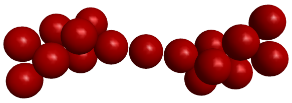 | 19B<br>Energy: -26.15380677<br>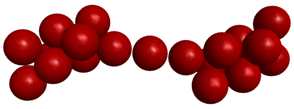 | 19C<br>Energy: -25.82448915<br>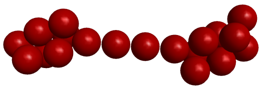 |
| 19D<br>Energy: -25.82448293<br>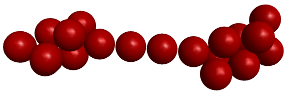 | 19E<br>Energy: -25.77530111<br>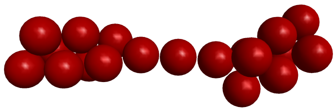 | 19F<br>Energy: -25.77528932<br>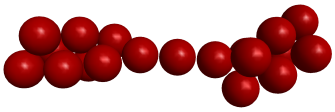 |
| 19G<br>Energy: -25.64763543<br>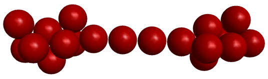 | 19H<br>Energy: -25.32602688<br>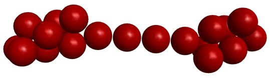 | 19I<br>Energy: -25.22691019<br>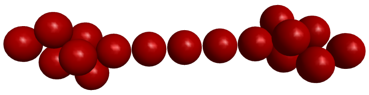 |

| | | |
|---|---|---|
| 19J<br>Energy: -25.22690894<br>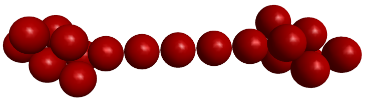 | 19K<br>Energy: -25.04443432<br>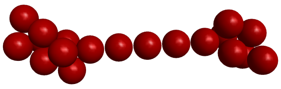 | 19L<br>Energy: -24.90223850<br>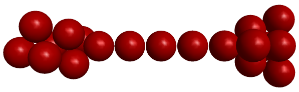 |

SET IIa

| | | |
|---|---|---|
| 19M<br>Energy: -24.33229396<br>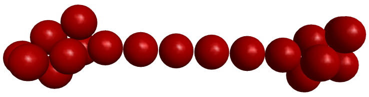 | 19N<br>Energy: -23.38694748<br>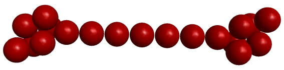 | 19O<br>Energy: -23.27318493<br>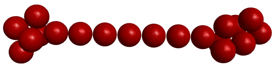 |
| 19P<br>Energy: -22.30356055<br>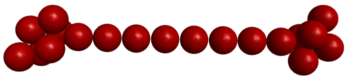 | | |

SET IIb’

| | | |
|---|---|---|
| 19Q<br>Energy: -21.20826570<br>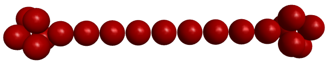 | 19R<br>Energy: -20.98809723<br>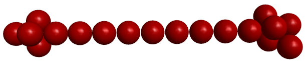 | 19S<br>Energy: -20.29574940<br>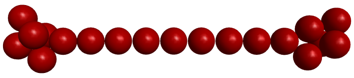 |
| 19T<br>Energy: -19.88718357<br>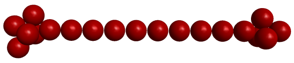 | 19U<br>Energy: -18.97362153<br>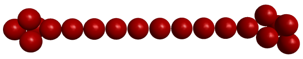 | 19V -18.56341295<br>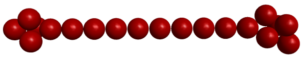 |

| | | |
|---|---|---|
| 19W<br>Energy: -16.78505999<br>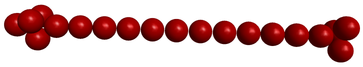 | | |

SET III

| | | |
|---|---|---|
| 19X<br>Energy: -11.10919223<br>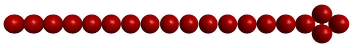 | 19Y<br>Energy: -10.26071989<br>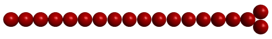 | 19Z<br>Energy: -9.87038493<br>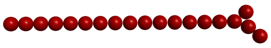 |
| 19ZA<br>Energy: -9.34036978<br>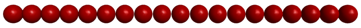 | | |

*N*=20

SET I

| | | |
|---|---|---|
| 20A<br>Energy:-27.29071051<br>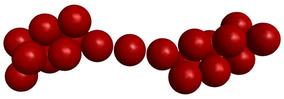 | 20B<br>Energy: -27.29066707<br>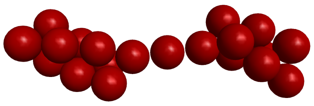 | 20C<br>Energy: -27.11649813<br>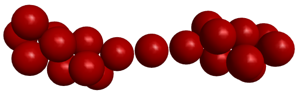 |
| 20D<br>Energy: -27.10925345<br>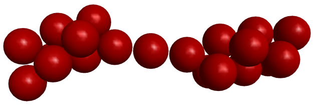 | 20E<br>Energy: -27.04871448<br>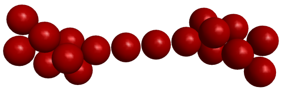 | 20F<br>Energy: -27.04870115<br>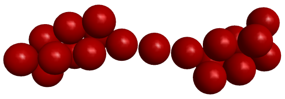 |

| | | |
|---|---|---|
| 20G<br>Energy: -26.99198881<br>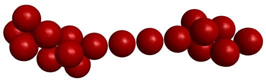 | 20H<br>Energy: -26.98188154<br>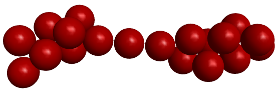 | 20I<br>Energy: -26.98177700<br>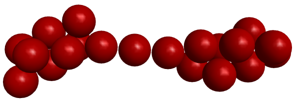 |
| 20J<br>Energy: -26.47371152<br>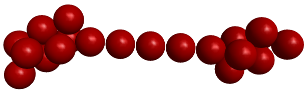 | 20K<br>Energy: -26.29580256<br>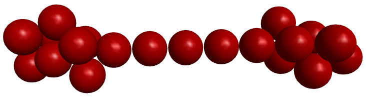 | |

SET IIa

| | | |
|---|---|---|
| 20L<br>Energy: -25.77286678<br>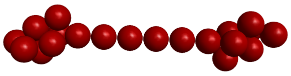 | 20M<br>Energy: -25.73793923<br>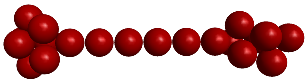 | 20N<br>Energy: -24.83288862<br>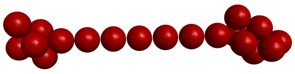 |
| 20O<br>Energy: -24.80228231<br>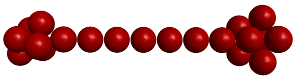 | 20P<br>Energy: -23.86736332<br>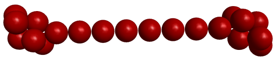 | 20Q<br>Energy: -22.77417092<br>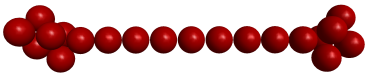 |

SET IIb

| | | |
|---|---|---|
| 20R<br>Energy: -21.67470745<br>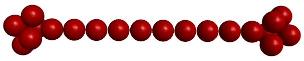 | 20S<br>Energy: -21.45411822<br>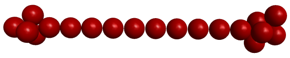 | 20T<br>Energy: -20.76142120<br>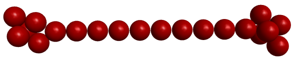 |

| | | |
|---|---|---|
| 20U<br>Energy: -20.35164942<br>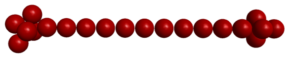 | 20V<br>Energy: -19.02713603<br>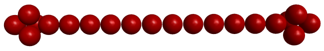 | |

SET III

| | | |
|---|---|---|
| 20W<br>Energy: -11.57245490<br>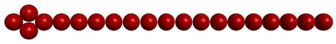 | 20X<br>Energy: -10.72399393<br>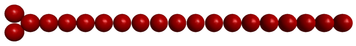 | 20Y<br>Energy: -10.33364733<br>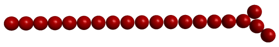 |
| 20Z<br>Energy: -9.80365022<br> | | |
